# Modern Medico-Psychology and Psychiatry

**Published:** 1894-04-28

**Authors:** T. S. Clouston

**Affiliations:** Lecturer on Mental Diseases, Edinburgh University, and Physician Superintendent Royal Asylum, Morningside


### Modern Medico-Psychology and Psychiatry.

By T. S. Clouston, M.D., Lecturer on Mental Diseases, Edinburgh University, and Physician Superintendent Royal Asylum, Morningside.

### IV.—LEGISLATIVE AND STATE MEASURES AND THEIR EFFECTS—(*continued*).

After various futile attempts a new Lunacy Act was passed in 1890. The chief alterations it made on the old law were the following. All private patients must under its provisions be sent to asylums or detained in private houses, not as before, on the order of a relative and two medical certificates, but as in Scotland, on a magistrate's order, and two such certificates. A provision was made for placing a patient under treatment on an "emergency" order of a relative and a single doctor's certificate for eight days, till the other certificate and the magistrate's order could be got. Patients may claim to see the committing magistrate. After they have entered asylums they must be visited by a Commissioner soon after admission. The order for their detention is not permanent, but must be renewed by the physician to the asylum, re-certifying the patient as being insane, and needing detention, at certain intervals. It made necessary certain wearisome and useless returns in regard to their patients on the part of those physicians, returns which make their lives a burden to some of them, and take up valuable time which should be given to medical work, so making them more of administrators and clerks and less of doctors than they were before. The Act was to some extent a sop to the dissatisfied, and was in the form of its chief new provisions a lawyer's and not a patient's or a Medical Act, as it should have been. One good thing it did at the urgent request of the medical profession, and that was to give proper security to the doctor who, after taking due care, signed a lunacy certificate against a subsequent action at law. By one of its provisions no new private asylums are to be allowed in England.

England, true to her feudal traditions, has made very special legislative provision for the care and supervision of the man who had property, and for the safe custody of his means. Parliament has passed many Acts for this purpose. It has a cumbrous, and in many cases costly but very efficient system of inquiry into his civil capacity by a "Master in Lunacy," aided sometimes by a jury. When thus found unfit to manage his affairs, a "committee of his person" and a "committee of his estate" are appointed. Three "Lord Chancellor's visitors," one legal and two medical, who visit him four times a year and report on him to the Chancellor, are the chief parts of the machinery provided by these Acts. He is in fact seen to with extraordinary minuteness. There are about one thousand such persons in England, and the yearly cost of originally inquiring into their incapacity, of visiting them, and of managing their property is far greater than the expense of the whole Lunacy Commission who have to visit and be responsible for the other 89,000. The Commission in fact costs the country £15,000 a year; while the Lord Chancellor's Lunacy Department costs it £23,000. The man with property costs £23 a year for guardianship, the man without means costs three and threepence. Why there should be a Lord Chancellor's Visitors' Department apart from the Commission is, like the distinctions between our Scotch Presbyterian sects, a thing that no one but a native can understand. The addition of the Lord Chancellor's Visitors to the staff of the Commission would add 50 per cent. to its inspecting power.

In effectual legislation for the mentally unsound Scotland lagged behind England for twelve years, though in making voluntary provision for their treatment she had before 1845 been distinctly in advance, taking her population and her poverty into account. The five chief towns of Scotland had, at the end of last century or early in this, each built a public or "Royal" Asylum, which in 1857, when the present Lunacy Act for Scotland was passed, accommodated 2,380 of the total number of 5,824 persons then known to be insane. But there existed by the side of this public provision, many abuses, which an American lady philanthropist, Miss Dix, brought energetically before the notice of the public and the authorities of the time in 1855. In 1856, a strong Royal Commission of enquiry, containing among its members two of the English Commissioners, was appointed, and on its report being issued in 1857, showing that great abuses existed, the present Act was at once passed that year. Under its provisions a "General Board of Lunacy" was appointed, with District Boards throughout the country, the General Board having the power to control, inspect and advise the latter, and to inspect the existing Royal Asylums. The General Board consisted of a chairman, two unpaid Commissioners, who have always been lawyers, and two paid Visiting Commissioners, who have always been medical men, with two deputy Medical Commissioners under them who are not members of the Board. The duty of the District Boards was to see that institutions for the treatment of the insane were provided out of the rates, whenever the Royal Asylums had not already provided accommodation. The Scotch Commission has worked remarkably well and to the satisfaction of the country during its thirty-six years of existence. Though working on the general lines of its English prototype, and beginning its building on the English foundation, its tone has been distinctly more medical than the English Commission. It has developed a system of boarding out quiet manageable pauper insane, under the guardianship of respectable country people who have comfortable cottages, whereby an individual family life is provided for them. In this way 20 per cent. of the pauper insane is now accommodated, thereby saving expensive enlargements to asylums, and making the patients far happier in a natural way than they would be in annexes to asylums for incurables. The abolition of small enclosed "airing courts," the "open door system" in asylums, and the detached "hospital" for those needing special bodily nursing, have been advocated and extended in Scotland through their influence. In Scotland, under the Court of Session Act of 1868, the property of the insane with means is amply protected by the simple, rapid, and inexpensive process of the appointment by a judge of the Court of Session of a *Curator Bonis*. The old process of "cognition," analagous to the English system of inquisition, is now almost abandoned.

In Ireland excellent asylums have been built in every district, and are visited by the two inspectors in lunacy, but their system is still tainted by the criminal idea of insanity, through two-thirds of all the new admissions to asylums each year being sent as "dangerous lunatics." But changes in the law are in contemplation there founded on the Scotch law and experience.

(*To be continued.*)

